# An investigation of the relationship and pathways of influence between body mass index, motor coordination, and health-related physical fitness index in preschool children

**DOI:** 10.3389/fpubh.2025.1585768

**Published:** 2025-07-17

**Authors:** Deqiang Zhao, Xiaoxiao Chen, Aoyu Zhang, Chunmiao Wang, Yibei Wang, Jin He, Jiaxin Chen, Haixia Hu, Xiaoni Tang, Aiying Zhang, Han Xiao, Yanfeng Zhang

**Affiliations:** ^1^National Physical Fitness and Science Fitness Center, China Institute of Sport Science, Beijing, China; ^2^Aiyumo Children and Youth Sports Health Research Institute, Weifang, Shandong, China; ^3^Changyi Experimental Kindergarten, Changyi City Education Bureau, Weifang, Shandong, China; ^4^Weifang National Fitness Service Center, Weifang Sports Bureau, Weifang, Shandong, China

**Keywords:** preschoolers, body mass index, motor coordination, physical fitness index, mediating effect

## Abstract

**Background:**

Movement development and motor ability are related to preschool children's physical health, and obesity is an increasingly serious problem in early childhood.

**Objective:**

The aim of this study was to analyze the mechanism of body mass index (BMI), motor coordination (MC), and physical fitness index (PFI) influencing pathways and to provide theoretical references for promoting the health management of preschool children.

**Methods:**

A total of 374 preschool children aged 3–6 years from a kindergarten in Weifang City, Shandong Province, China, were recruited to this study using a stratified random sampling method. We evaluated the motor coordination of these preschool children through the Movement Assessment Battery for Children, Second Edition (MABC-2) and assessed the physical fitness index of their sports performance through physical fitness tests such as standing long jump and continuous two-foot jumping. Pearson correlation analysis, network analysis, and hierarchical regression analysis were used to verify the interaction between BMI, MC, and PFI at each level. Finally, the bootstrap method was used to test the mediating effect of motor coordination.

**Results:**

The results of the network analysis indicated that body mass index negatively affected preschoolers' motor coordination and physical fitness index. The stratified regression results indicated that body mass index negatively influenced physical fitness index (*p* < 0.01) and motor coordination positively influenced it (*p* < 0.01). Movement coordination played a mediating role (95% CI = [−0.077, −0.015]).

**Conclusions:**

This study, based on cross-sectional data analysis, reveals the interrelationship between BMI, MC, and PFI in preschool children. BMI is significantly negatively correlated with MC and PFI, suggesting that lower MC and PFI may be associated with higher BMI levels. MC mediates the relationship between BMI and PFI, and improving motor coordination can weaken the negative association between BMI and PFI. Therefore, in promoting the sports health of preschool children, instead of simply increasing the amount of physical activity, training in motor coordination can be added to improve their sports performance.

## 1 Introduction

With the continuous improvement in socio-economic conditions and changes in modern lifestyles, the health of preschool children has attracted increasing global attention (1, 2). Early childhood is a critical period in human development during which motor development and motor abilities are not only closely linked to physical health but also play a pivotal role in shaping cognitive abilities, social skills, academic achievement, and future psychological wellbeing (3, 4). During this stage, rapid changes in body morphology significantly influence motor development and competence (5). Although motor skills vary throughout childhood, they remain foundational to children's overall physical health (6, 7). For instance, both physical fitness and physical fitness index (PFI) have been found to be associated with lower risks of obesity and better cardiorespiratory and muscular fitness (8, 9). Previous research has consistently demonstrated positive associations between motor skills and components of physical fitness, such as balance, coordination, speed, and agility (10), suggesting that motor development and coordination can serve as critical indicators of children's physical fitness.

Body mass index (BMI) is widely used as a measure to evaluate overweight and obesity. Among preschool children, BMI is not only an important indicator of physical health but also reflects nutritional status and lifestyle behaviors (11). The global rise in childhood obesity poses increasing health risks, including heightened chances of developing chronic conditions such as cardiovascular disease and diabetes, and may also impair social functioning and mental health (12). Based on BMI values, children are typically categorized as normal weight, overweight, or obese, with the last group being more prone to movement difficulties and poor motor coordination (13, 14). Hence, BMI is strongly associated with children's health, especially in the context of overweight and obesity (15, 16).

Motor coordination (MC) is fundamental to the development of motor abilities. As a core motor quality, it reflects the integration of various neuromuscular resources and is considered a key prerequisite for all forms of physical activity (17–19). It refers to the ability to harmoniously control body parts during movement, commonly seen in activities such as running, jumping, and throwing (20). MC not only influences daily function and PFI but also reflects the development of the central nervous system (21). During the preschool years, it continues to evolve, and insufficient physical activity or excessive BMI may disrupt this process, leading to suboptimal motor development and related issues in physical and psychological health (22, 23).

The health-related physical fitness index (PFI) provides a comprehensive measure of children's physical condition, typically encompassing cardiorespiratory endurance, muscular strength, and flexibility (24, 25). Higher fitness levels are associated with stronger immunity, healthier musculoskeletal and neurological development, and better psychosocial outcomes (26). Conversely, poor fitness, particularly in children with obesity, is often accompanied by low cardiorespiratory capacity, weak muscle strength, and high levels of fatigue, potentially laying the groundwork for chronic diseases later in life (27). Fitness also influences children's mobility, social participation, and emotional regulation (28).

In multidimensional evaluations of children's health, strong associations have been reported among BMI, motor coordination, and physical fitness (29, 30). Excessive BMI is often linked to poorer MC and lower PFI, as children with obesity typically demonstrate reduced endurance, flexibility, and coordination (31). These deficits may further impair physical health, psychological resilience, and long-term quality of life (32, 33). Therefore, exploring the interrelationships between BMI, MC, and PFI in preschoolers holds significant theoretical and practical value.

**Figure 1:**
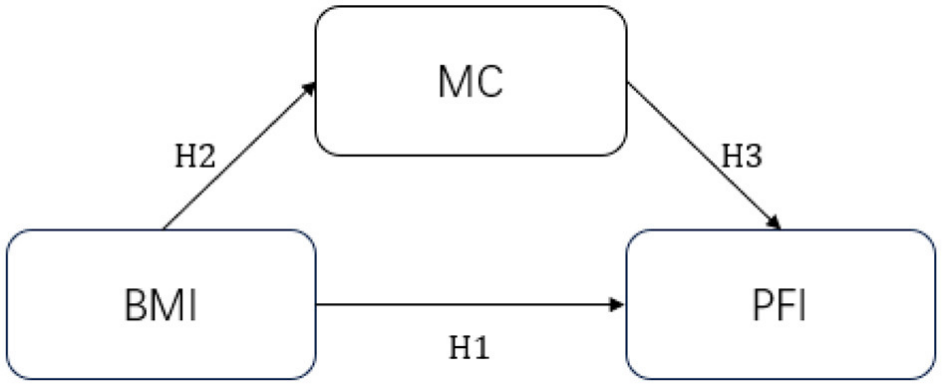
Path relationship assumptions.

The present study was designed to test the associations between BMI, MC, and PFI, with a specific focus on the potential mediating role of motor coordination. By constructing a research hypothesis framework (Figure 1) and analyzing the aforementioned variables, this study addresses a gap in the existing literature, as few prior studies have examined this relationship using preschool-aged samples. The findings from this study are expected to contribute to the theoretical understanding of early childhood health and inform the development of targeted interventions and individualized strategies for promoting physical health in preschool children.

## 2 Materials and methods

### 2.1 Participants

A total of 374 preschool children were recruited from a kindergarten in Weifang City, Shandong Province, China, using a stratified random sampling method. The experimental period was from October 2023 to January 2024. After excluding 16 children with incomplete data, 358 children (181 boys and 177 girls) were included in the final analysis. All parents or legal guardians of the participants signed an informed consent form before testing. This study was conducted in accordance with the Declaration of Helsinki and was approved by the Institutional Review Board of the Chinese Institute of Sport Science (No. CISSLA20230110).

### 2.2 Measurements

#### 2.2.1 Body mass index

Height and weight were measured according to the Chinese National Physical Fitness Standard Manual for Preschoolers (CPFS-preschool), and BMI was calculated (34).

#### 2.2.2 Motor coordination

The Movement Assessment Battery for Children, Second Edition (MABC-2) is used to effectively assess the development of motor coordination in preschool children (35). Since 2016, China has had a Chinese version of the copyright and norms, which is an important reference value for the assessment of children's motor ability (36). The normative assessment is divided into three age groups: 3–6 years old, 7–10 years old, and 11–16 years old. Each age group has eight tasks attributed to three dimensions: manual dexterity, body coordination, and balance. These three abilities serve as the basic components of a child's level of motor development as he or she grows and develops, allowing for a comprehensive assessment of all aspects of a child's motor development. It takes about 30–40 min for children to complete the assessment. The test scores are entered into an officially licensed system, and the test is converted from raw item scores to standardized scores, taking into account age and gender. Finally, the scores are standardized to a composite standard score that reflects overall motor coordination.

#### 2.2.3 Physical fitness index

Before the test, the requirements were demonstrated and explained to the children, and the preschoolers were tested on six items: standing long jump, tennis ball throw, 10 m run, 15 m run around obstacles, sitting forward bend, and walking on balance beam. The physical fitness test was conducted according to the physical fitness test rules in the Chinese National Physical Fitness Standard Manual for Preschoolers (CPFS-preschool). The scores of the six physical fitness indicators were standardized according to gender and age, and z-scores were calculated. Finally, the z-scores of the six tests were summed to evaluate the preschoolers' PFI.

### 2.3 Statistical methods

First, descriptive statistical analysis was used to observe sample characteristics. For continuous variables showing a normal distribution, the independent samples *t*-test was used to assess gender differences. For variables showing non-normal distribution, the Mann–Whitney *U*-test was used to compare median statistics. Pearson correlation analysis was used to initially analyze the correlation between body mass index, motor coordination, and PFI. To further understand the relationship between the three, the interaction between them was explored through R language network analysis. To more clearly show the effect of the independent variables on the dependent variable, a hierarchical linear regression method was used, and two regression models were constructed for analysis. Model 1 incorporated the demographic control variables age and gender. Model 2 incorporated body mass index and motor coordination standardized scores. Finally, the mechanistic pathways were examined using Model 4 from Hayes's (2017) PROCESS macro (37), with bootstrap mediation analysis (5,000 resamples, 95% CI) applied to test the mediating effects among BMI, motor coordination, and PFI. All statistical procedures were executed in SPSS 27.0 and R version 4.3.0.

## 3 Results

### 3.1 Sample characteristics

Table 1 displays the baseline characteristics and descriptive statistics of demographic variables, motor coordination, physical fitness index, and so on in the preschooler sample.

**Table 1:** Sample characteristics.

**Variables**	**Total**	**Boys**	**Girls**
Demographic characteristics	358	181	177
Age	4.46 ± 0.79	4.46 ± 0.80	4.46 ± 0.78
Body mass index (BMI)	18.86 ± 3.60	19.45 ± 3.93	18.26 ± 3.14
**Motor coordination (MC)**			
Fine hand movements	9.06 ± 2.50	8.49 ± 2.51	18.26 ± 3.14
Physical coordination	11.11 ± 2.50	10.99 ± 2.52	108.76 ± 13.63
Dynamic and static balance	11.10 ± 2.35	10.72 ± 2.78	10.72 ± 2.28
**Athletic Performance**			
Physical fitness index (PFI)	0.72 ± 3.49	10.72 ± 2.28	0.84 ± 3.21

### 3.2 Correlation between body mass index, motor coordination, and physical fitness index

Table 2 presents a correlation matrix of key variables. Pearson correlation analysis demonstrated that, among the key independent variables, BMI was significantly negatively correlated (*p* < 0.01) with MC and BMI. In addition, the mediator variable MC was significantly positively correlated with PFI (*p* < 0.01). Based on the results of the correlation analysis, we will preliminarily verify the relationship between BMI and MC and PFI.

**Table 2:** Correlation analysis of key variables.

**Variables**	**Correlation indicator**	**BMI**	**PFI**	**MC**
BMI	*r*	1		
	*p*			
PFI	*r*	−0.271	1	
	*p*	0.001		
MC	*r*	−2.232	0.312	1
	*p*	0.001	0.001	

### 3.3 Network analysis of body mass index, movement coordination, and physical fitness index

**Figure 2:**
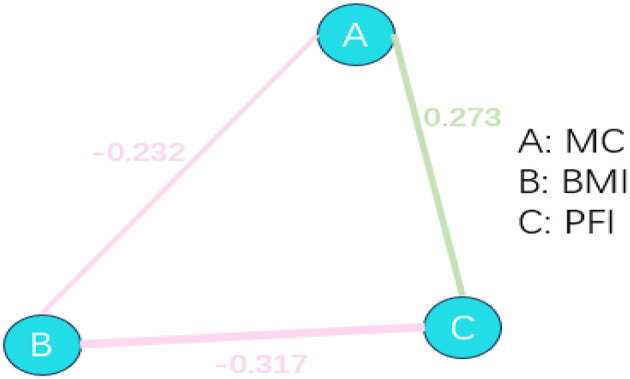
Structure of the network of BMI, MC, and PFI relationships. Red lines indicate negative correlation, green lines indicate positive correlation, and the thickness of the lines represents the size of the correlation.

**Figure 3:**
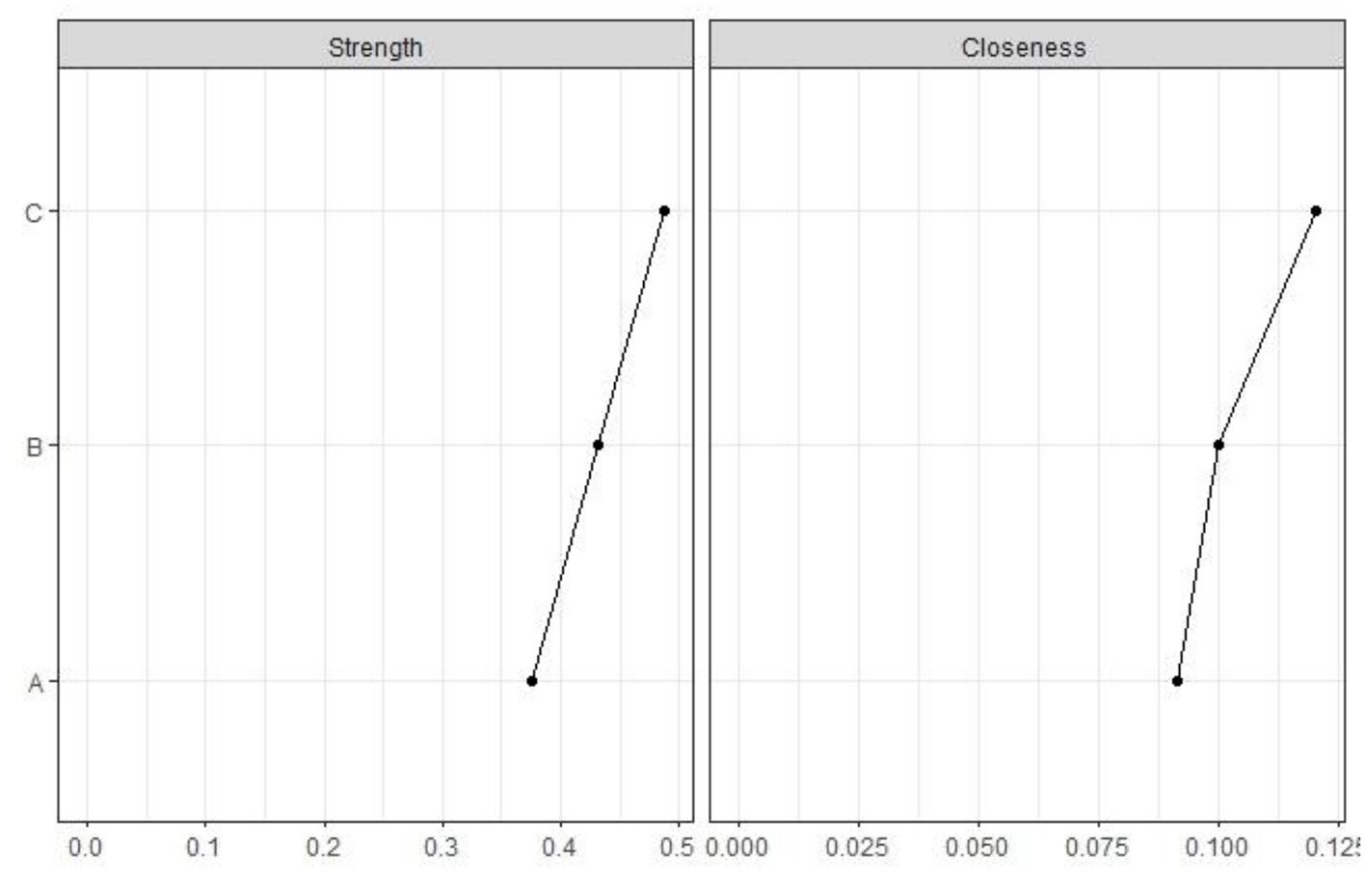
Standard EI index for network elements.

The interaction between BMI, MC, and PFI was further verified through R language network analysis. A network graph with three nodes and three edges was constructed. As shown in Figure 1, BMI, MC, and PFI interacted with each other. BMI significantly and negatively affected MC (*r* = −0.232) and PFI (*r* = −0.317). MC significantly positively influenced the relationship with PFI (*r* = 0.273). As seen in Figure 2, the network centrality estimation shows that PFI centrality estimation is the strongest (C, EI = 0.485). It plays an important role in the network, and its activation has the strongest effect on other nodes in the network (Figures 2 and 3).

**Figure 4:**
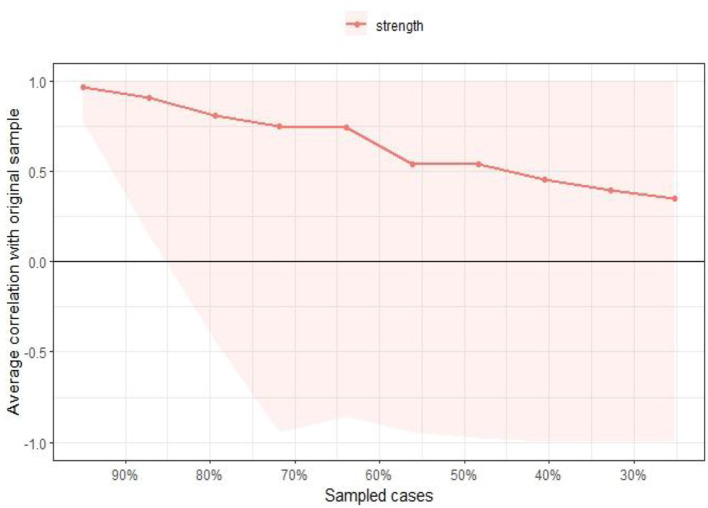
Bootstrap test for CS coefficients of network nodes.

We tested the stability of the network centrality index using the bootstrap method (nBoots = 1,000), with intensity being the most accurately estimated centrality index. The CS coefficient for EI centrality is 0.425, indicating that the results for the network centrality index are within acceptable limits (Figure 4).

### 3.4 Multiple linear regression analysis of body mass index, motor coordination, and physical fitness index

To further validate the relationship between BMI, MC, and PFI, two models were developed using hierarchical linear regression. Model 1 incorporated age and gender as control variables, and the results showed that they did not significantly affect the PFI. Model 2 incorporated the key variables BMI and MC, and the results showed that BMI significantly and negatively influenced the PFI of preschoolers (β = −0.317, *p* < 0.01). MC significantly and positively influenced children's PFI (β = 0.244, *p* < 0.01; Table 3).

**Table 3:** Stratified linear regression analysis.

**Variables**	**Model 1**			**Model 2**		
	***b***	**SE**	β	***b***	**SE**	β
Sex	0.063	0.519	0.009	−0.497	0.495	−0.068
Age	−0.387	0.307	−0.089	0.536	0.327	0.124
BMI				−0.279	0.065	−0.317^**^
MC				0.334	0.097	0.244^**^
*R* ^2^		0.008			0.151	

^**^p < 0.01.

### 3.5 Analysis of mediating effects of motor coordination

**Figure 5:**
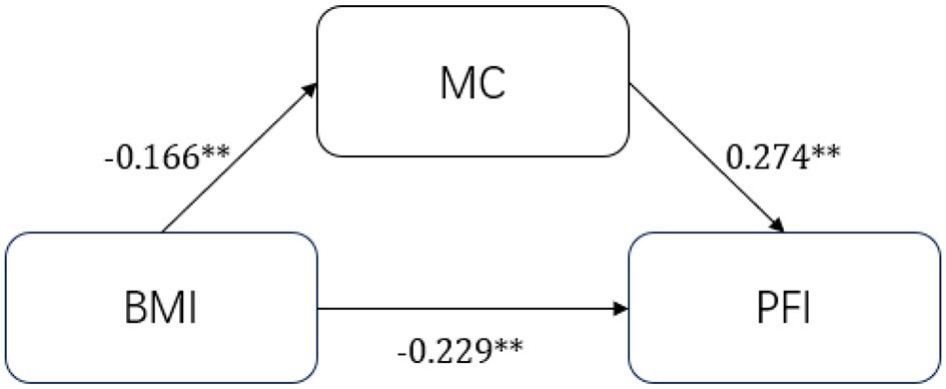
Mediation model of BMI effect on PFI. **, p < 0.01.

In this section, we will further investigate the pathways of BMI and the effects on PFI in preschoolers. In the theoretical analysis section, we determined the internal logic between BMI, MC, and PFI. In the correlation analysis, we found that there is a significant correlation between them. Using network analysis, we verified that there is an interaction effect relationship among them. In the regression analysis, after adding gender and age as control variables, BMI negatively affects preschool children's PFI, whereas motor coordination positively affects it. On this basis, this section implements a path mechanism for the effect of BMI on the PFI. We constructed a mediation test model with PFI as the dependent variable, BMI as the independent variable, and MC ability as the mediator variable. The estimation results of the path coefficients are shown in Figure 4, and the results of the chain mediation effect analysis are shown in Table 4. To clearly demonstrate the interrelationships between variables, we report standardized path coefficients in this section. We repeated the sampling 5,000 times using the bootstrap method to analyze the main and chain mediation effects. The results showed a path indirect effect of −0.045 (95% CI = [−0.077, −0.015]) with a contribution of 16.55% for MC as the mediating variable, a direct effect of −0.229 (95% CI = [−0.346, −0.112]) with a contribution of 83.45% for BMI on PFI. The total effect of the mediated pathway effect through movement coordination was −0.274 (95% CI = [−0.389, −0.159]). The mediated effect of preschoolers' BMI affecting PFI through MC was established (Figure 5, Table 4).

**Table 4:** Bootstrap analysis for significance test of mediation effect.

**Impact paths**	**Effect**	**BootSE**	**BootLLCI**	**BootULCI**	**Contribution (%)**
Total	−0.274	0.058	−0.389	−0.159	
BMI-PFI	−0.229	0.059	−0.346	−0.112	83.45%
BMI-MC-PFI	−0.045	0.158	−0.077	−0.015	16.55%

## 4 Discussion

This study investigated the relationship between body mass index, movement coordination, and PFI in preschool children. It also examined children's body shape, basic movement, and physical fitness development as a basis to further explore the interactive influence relationship and path mechanism between preschool children's body shape, movement coordination, and PFI. Moreover, it explored the pathways and mechanisms of the relationships among body mass index, motor coordination, and PFI in preschool children through correlation analysis, network analysis, regression modeling, and mediation effects at a hierarchical level.

### 4.1 Relationship between body mass index, motor coordination, and physical fitness index

Body form has a significant impact on preschoolers' fundamental motor skills (FMS), influencing both their ability to perform basic movements and PFI (e.g., strength, flexibility, and coordination) (38, 39). FMS are foundational skills that enable preschoolers to participate in various physical activities and are typically exemplified by movements such as running, jumping, and throwing (40). One of the key components underlying FMS development is motor coordination, which reflects the ability of different body parts to work together efficiently and is often demonstrated through movements requiring balance, timing, and control. Our results showed that a higher BMI was significantly associated with poorer MC and a lower PFI, indicating that increased body mass may hinder physical coordination and performance in preschoolers. Moreover, MC ability was positively associated with the PFI, suggesting that better coordination supports improved PFI. These findings are in line with previous studies showing that motor coordination is negatively related to weight status and positively related to physical activity (PA) and perceived physical competence in both cross-sectional and longitudinal studies (41–43). Together, these results highlight the critical role of body form in shaping preschoolers' motor coordination and physical fitness, providing indirect evidence of the interconnectedness among body form, coordination ability, and PFI.

To further validate this triadic relationship, we conducted a network analysis using R to examine the interactive effects of BMI, MC, and PFI. The results showed that an increase in BMI in preschool children was significantly associated with a decrease in motor coordination and PFI, which was consistent with a previous study (44). This association may be due to the combined effects of reduced neuromuscular control efficiency (e.g., increased joint load caused by obesity) and psychosocial interactions (e.g., motor withdrawal behavior). Additionally, we observed that preschoolers with better motor coordination exhibited significantly higher physical fitness, which aligns with the findings of Laukkanen et al. (45), who reported a positive correlation between moderate-to-vigorous physical activity (MVPA) and MC, particularly among boys. This supports the hypothesis that MC and PA form a bidirectional facilitative loop: children with higher MC are more likely to engage in high-intensity activities, and frequent motor participation, in turn, enhances neuromuscular control, thereby reinforcing MC over time. This mechanism may explain the role of coordination in enhancing PFI observed in our study. Moreover, observed gender differences, for example, greater sensitivity to the negative impact of BMI among girls, suggest that psychosocial maturity or developmental factors may moderate the relationship between MC and PFI. Future research should explore these moderating effects across multiple dimensions, incorporating factors such as family support and physical activity environments.

To further substantiate these findings and control for potential confounders, we conducted a hierarchical regression analysis by including age and sex as covariates. The results reaffirmed that higher BMI was associated with a lower PFI, while higher motor coordination ability predicted better physical fitness outcomes. These results remained robust even after accounting for age and sex, supporting the generalizability of the observed relationships across subgroups. Furthermore, previous studies have reported an inverted U-shaped (parabolic) relationship between BMI and motor coordination, where both underweight and overweight status were associated with reduced MC, alongside a concurrent increase in BMI (46). This non-linear pattern suggests that optimal motor coordination may be achieved within a healthy BMI range, reinforcing the importance of balanced body composition in early childhood motor development.

The increasingly negative correlation between motor coordination and BMI observed throughout childhood underscores the importance of allocating sufficient time for both free play and structured physical activities to support the development of motor coordination (47). Research has also demonstrated that higher BMI is negatively associated with physical fitness components such as flexibility and muscular strength in early childhood (48). The impact of BMI on PFI during this developmental stage is multifaceted, involving factors such as excessive body weight, reduced muscle strength, impaired coordination, and diminished cardiorespiratory fitness (49). Typically, a higher BMI implies overweight or obesity, which increases the physical load during movement, resulting in diminished coordination and endurance and, consequently, reduced PFI (50). Children with obesity often struggle with tasks requiring balance, agility, and strength, and excessive body fat combined with reduced muscular strength can further hinder motor skill development, elevate fatigue, and prolong recovery time (51). As BMI adversely affects motor coordination, it indirectly yet significantly impairs overall PFI. These associations reveal an interrelated and cyclical relationship among BMI, motor coordination, and PFI: lower BMI is associated with enhanced coordination, which subsequently facilitates better PFI, whereas higher BMI compromises coordination and endurance, leading to reduced athletic outcomes (52).

### 4.2 Analysis of mediating effects of Motor coordination

To further clarify the pathway through which BMI affects PFI in preschool children, we conducted a mediation analysis to test the theoretical framework derived from previous studies and our empirical findings. The results confirmed that MC serves as a significant mediator in the relationship between BMI and the PFI. Specifically, BMI exerted a negative effect on the preschoolers' PFI by impairing their coordination ability. Motor coordination partially attenuated the adverse impact of high BMI on physical fitness.

While earlier studies have primarily examined the pairwise associations among BMI, MC, and PFI, our analysis reveals their interconnected mechanism. Our findings indicate that BMI negatively influences both MC and PFI, whereas MC positively influences PFI. The presence of better motor coordination appears to buffer the negative effect of BMI on PFI, suggesting a mediating role. This mediating mechanism may be explained by the fact that excess body weight imposes a greater physical burden during movement, leading to reduced coordination, particularly in activities that demand balance, agility, and precise control of movement (53). In contrast, children with higher coordination can better regulate body posture, execute complex motor tasks with greater efficiency, and display improved motor outcomes (54). Good coordination enhances movement efficiency, lowers energy cost, and increases precision and flexibility.

Thus, the mechanism by which BMI influences PFI through motor coordination reflects a cascading process: increased BMI leads to impaired neuromuscular control and reduced coordination, which in turn diminishes PFI (55). On the other hand, improving weight management may enhance coordination and thereby improve overall physical fitness in preschoolers (56). Taken together, these results underscore the importance of targeting motor coordination as a key intervention point in the health promotion of preschool children. Rather than solely focusing on increasing the amount of physical activity, efforts should also prioritize enhancing fundamental motor skills and coordination. This dual approach can optimize children's motor development and PFI, contributing to long-term health outcomes (57).

#### 4.2.1 Strengths and limitations

This study explored the relationship between BMI, MC, and PFI in preschool children using cross-sectional data and various methods. However, the research has some limitations: (1) the study sample is geographically limited, as the participants were from a kindergarten in Shandong Province, China, which may restrict the generalizability of the findings. Future research should aim to expand the sample size to include different regions and socio-economic backgrounds; (2) as a cross-sectional design was used, causal relationships cannot be inferred. Longitudinal or experimental studies are needed to confirm the temporal sequence of the effects and the robustness of the mediating role of MC over time; and (3) although this study controlled for age and gender, other potentially important factors, such as physical activity levels, nutrition, and sleep, were not included. These factors may be important behavioral influences on BMI, MC, and PFI and are closely related to children's BMI.

Therefore, future research should incorporate comprehensive measures of nutrition, physical activity, and sleep in longitudinal or experimental designs. For example, dietary recall questionnaires, objective physical activity monitoring (such as accelerometers), and sleep assessments (such as sleep logs) could be used to control for the impact of confounding factors on the results. Prospective cohort studies or intervention experiments would be more likely to clarify the causal pathways between lifestyle behaviors, BMI, MC, and PFI. Including these variables as covariates or intervention targets in analytical models will help more accurately identify the independent effects of BMI, MC, and PFI, providing a more scientific and effective evidence base for research on children's health and development.

## 5 Conclusions

This study, based on cross-sectional data analysis, reveals the interrelationship between BMI, MC, and PFI in preschool children. The conclusions are as follows: (1) BMI is significantly negatively correlated with MC and PFI, suggesting that lower MC and PFI may be associated with higher BMI levels; and (2) MC mediates the relationship between BMI and PFI, and improving MC can weaken the negative association between BMI and PFI. Therefore, in future efforts to promote physical health management in preschool children, it is important to focus on nutrition intake to prevent excessive body weight from negatively affecting motor abilities. Additionally, for children with overweight or obesity, in addition to simply increasing physical activity levels, attention should be given to the development of fundamental motor skills (such as fine hand movements and gross motor skills).

## Data Availability

The data analyzed in this study is subject to the following licenses/restrictions: The data contains the privacy of minors and is not available to the public. Requests to access these datasets should be directed to Yanfeng Zhang, zhangyanfeng@ciss.
